# Factors associated with loss to follow up among HIV-exposed children: a historical cohort study from 2000 to 2017, in Porto Alegre, Brazil

**DOI:** 10.1186/s12889-022-13791-9

**Published:** 2022-07-26

**Authors:** Karen da Silva Calvo, Daniela Riva Knauth, Bruna Hentges, Andrea Fachel Leal, Mariana Alberto da Silva, Danielle Lodi Silva, Samantha Correa Vasques, Letícia Hamester, Daila Alena Raenck da Silva, Fernanda Vaz Dorneles, Fernando Santana Fraga, Paulo Ricardo Bobek, Luciana Barcellos Teixeira

**Affiliations:** 1 Graduate Studies Program in Public Health, Federal University of Rio Grande Do Sul, Porto Alegre, RS 90620-110 Brazil; 2 Department of Social Medicine, Federal University of Rio Grande Do Sul, Porto Alegre, RS Brazil; 3 Graduate Studies Program in Epidemiology, Federal University of Rio Grande Do Sul, Porto Alegre, RS Brazil; 4 Graduate Studies Program in Public Policy, Federal University of Rio Grande Do Sul, Porto Alegre, RS Brazil; 5 Professional Master’s in Family Health, Federal University of Rio Grande Do Sul, Porto Alegre, RS Brazil; 6 School of Nursing, Federal University of Rio Grande Do Sul, Porto Alegre, RS Brazil; 7 General Directorate of Health Surveillance, Porto Alegre, RS Brazil; 8 Department of Public Health, Federal University of Rio Grande Do Sul, Porto Alegre, RS Brazil

**Keywords:** HIV/AIDS, Children exposed to HIV, Lost to follow-up, Mother-to-child transmission of HIV

## Abstract

**Background:**

There are many inequalities in terms of prevention and treatment for pregnant women with HIV and exposed children in low and middle-income countries. The Brazilian protocol for prenatal care includes rapid diagnostic testing for HIV, compulsory notification, and monitoring by the epidemiological surveillance of children exposed to HIV until 18 months after delivery. The case is closed after HIV serology results are obtained. Lost to follow-up is defined as a child who was not located at the end of the case, and, therefore, did not have a laboratory diagnosis. Lost to follow-up is a current problem and has been documented in other countries. This study analyzed factors associated with loss to follow-up among HIV-exposed children, including sociodemographic, behavioral, and health variables of mothers of children lost to follow-up.

**Methods:**

This historical cohort study included information on mothers of children exposed to HIV, born in Porto Alegre, from 2000 to 2017. The research outcome was the classification at the end of the child's follow-up (lost to follow-up or not). Factors associated with loss to follow-up were investigated using the Poisson regression model. Relative Risk calculations were performed. The significance level of 5% was adopted for variables in the adjusted model.

**Results:**

Of 6,836 children exposed to HIV, 1,763 (25.8%) were classified as lost to follow-up. The factors associated were: maternal age of up to 22 years (aRR 1.25, 95% CI: 1.09–1.43), the mother’s self-declared race/color being black or mixed (aRR 1.13, 95% CI: 1.03–1.25), up to three years of schooling (aRR 1.45, 95% CI: 1.26–1.67), between four and seven years of schooling (aRR 1.14, 95% CI: 1.02–1.28), intravenous drug use (aRR 1.29, 95% CI: 1.12–1.50), and HIV diagnosis during prenatal care or at delivery (aRR 1.37, 95% CI: 1.24–1.52).

**Conclusion:**

Variables related to individual vulnerability, such as race, age, schooling, and variables related to social and programmatic vulnerability, remain central to reducing loss to follow-up among HIV-exposed children.

## Background

Vertical HIV transmission in children can be avoided through prevention and treatment for pregnant women with HIV and exposed children [1, 2]. In 2020 85% [63%–98%] of pregnant women living with HIV had access to antiretroviral drugs to prevent transmission of HIV to their children [3]. This percentage, however, varies according to the weaknesses related to prenatal and postnatal care of health systems, especially in developing countries [4–6].

In Brazil, from 2000 to June 2020, 134,328 HIV-infected pregnant women were notified [7]. As for the rate of HIV detection in pregnant women, there was an increase of 21.7% over ten years: in 2009, 2.3 cases/thousand live births were registered, and, in 2019, this rate increased to 2.8 cases/thousand live births. Compared to other Brazilian state capitals, Porto Alegre, the location of this study, is the capital with the highest detection rate, with 17.6 cases/thousand live births recorded in 2019, a rate six times higher than the national rate. The AIDS detection rate in children under five years of age has been used as a proxy indicator for monitoring vertical HIV transmission. In 2019, this rate in Brazil was 1.9 cases/100,000 inhabitants. Porto Alegre is the fourth capital with the highest rate, with 7.2 cases/100,000 inhabitants [7].

Reducing the number of children infected with HIV and offering 95% treatment coverage is among the 95–95-95 goals that aim to prevent 5.9 million infections in children up to 15 years of age by 2030 [8]. Over the years, many actions have been documented in this area, especially in prenatal care, among which are the intensification of educational activities, the provision of rapid tests, the supply of antiretroviral therapy, and supplies of substitute inputs for breastfeeding [9–12]. Despite this, early diagnosis remains one of the main challenges [13–15]. The absence of a confirmatory test in children precludes the timely start of ART [16–18].

There are many inequalities prevention and treatment for pregnant women with HIV and exposed children in low and middle-income countries [6, 18–21]. In Brazil, ART is universally distributed by the public health system. Therefore, an epidemiological surveillance system with complete information on children exposed to HIV would significantly increase the ability to target services to those that need them.

The Brazilian protocol for prenatal care includes rapid diagnostic testing for HIV in the prenatal period with compulsory notification. Compulsory notification is a standardized document filled out during the pregnant woman’s visit when the HIV test result is positive. In the postnatal period, children exposed to HIV are monitored by epidemiological surveillance until 18 months after delivery [22]. The case is closed after HIV serology results are obtained. All this data is stored in a single database. Lost to follow-up is defined as a child who was not located at the end of the case and, therefore, did not have a laboratory diagnosis. Lost to follow-up is a current problem documented in other countries [18, 23–26].

In the city of Porto Alegre, approximately 400 children exposed to HIV are monitored annually. Case closure as lost to follow-up is not uncommon. Ignoring the HIV status of exposed children makes it impossible to start early interventions, contributing to infant morbidity and mortality [1, 27–29]. This study analyzed the factors associated with the children exposed to HIV being lost to follow-up (sociodemographic, behavioral, and health variables of mothers lost to follow). All sociodemographic and behavioral variables available in the National Surveillance System related to pregnant women living with HIV were investigated.

We believe that this analysis can contribute to developing more systematic monitoring strategies, strengthening epidemiological surveillance in Porto Alegre, the Brazilian state capital with the highest rate of HIV detection in pregnant women.

## Methodology

A historical cohort study was conducted in 2021. We extracted data from a national registry system from 2000 to 2017, characterizing the follow-up period.

## Population

The study uses a national database with information on pregnant women living with HIV and children exposed to HIV who were born in Porto Alegre. In the study scenario, the investigation of the child’s exposure to HIV ends up to 24 months after birth. This historical cohort study included information on pregnant women living with HIV and the outcome of the child’s exposure to HIV. For diagnosis in children under 18 months of age, the following tests are available: molecular test to quantify HIV-RNA/HIV viral load (CV-HIV) and detect HIV proviral DNA. The first CV-HIV collection must be performed immediately after birth. Any test whose result shows detectable CV-HIV, regardless of the viremia value, will require an immediate CV-HIV collection. The second test, if the first CV-HIV is undetectable, will be collected at 14 days of age. Unconfirmed cases should continue under investigation, with CV-HIV collections at two and eight weeks after the end of antiretroviral prophylaxis. An anti-HIV test should be performed at 12 months and, if positive, repeated at 18 months. In children with CV-HIV below 5,000 copies/mL or discordant results, proviral DNA, which has high specificity, should be used. The child will be considered HIV-infected if there are two detectable CV-HIV results above 5,000 copies/mL or positive proviral DNA [30].

In Porto Alegre, the deadline for closing the case runs from 18 to 24 months. The child is considered lost to follow-up at 24 months of follow-up without the completion of the laboratory diagnosis when the case is closed [31]. The unavailability of information is usually due to the lack of health care for the child in health units, at an age in which children should get regular check-ups.

## Data collection and variables

The epidemiological surveillance service is responsible for registering the information of pregnant women living with HIV, as well as monitoring and updating the health outcome of children exposed to HIV. In Brazil, such data are stored in a system called “Notifiable Diseases Information System” (SINAN), fed by compulsory notification. The database made available by the epidemiological surveillance service included all children exposed to HIV notified in the city of Porto Alegre with their respective updated results.The research data were collected by two trained researchers, during a six-month period in the year 2020, directly from SINAN. There were cases with missing information on race/color. To look for this information, other information systems that are used for medical records were accessed, such as the Laboratory Examination Control System (SISCEL), the Logistic Control System for Medicines (SICLOM), the electronic medical record of the primary care user (E-SUS) and the SUS User Registration System (CADSUS). The sociodemographic data presented refer to pregnant women at the time they were notified of HIV by the health services. Sociodemographic (age, race/color, and schooling), behavioral (lifetime injecting drug use), and health-related (time of HIV diagnosis, beginning of prenatal care, and use of ART during childbirth) information of pregnant women were all collected in the system in order to analyze possible associated factors (independent variables). The outcome of the child’s exposure was obtained using the same system (move to a different city, death, abortion, HIV positive, HIV negative, or lost to follow-up). Cases the pregnancy resulted in abortion, cases the children died within 24 months of follow-up, and cases moved to a different city were excluded because they did not complete the follow-up. The dependent variable was lost to follow-up. In terms of the variables, schooling was categorized as “up to three” years of study (including women who never attended school), “between four and seven” years of study, and “eight years or more” years of study. The information on the moment of HIV diagnosis for the pregnant woman was already grouped in the original data into two categories: (i) before prenatal care, or (ii) during prenatal care or at delivery. This was therefore used with these two categories in the statistical analysis.

The study’s outcome was the closure status of children exposed to HIV (yes or not) cases. Lost to follow-up is defined as a child who was not located at the end of the case, and, therefore, did not have a laboratory diagnosis.

## Statistical analysis

Statistical analysis was performed using SPSS Software, version 22.0. Absolute numbers and frequencies were used for sample description. Comparisons were made either using the homogeneity of proportions test based on Pearson’s or Fisher’s chi-square statistics and standardized residuals. The level of significance adopted was 5%.

The association between predictors and the child’s outcome as a loss to follow-up was investigated using the Poisson regression model. Relative Risk calculations were performed in unadjusted and adjusted models. The level of significance used was *p* < 0.20 in the unadjusted model for insertion of variables in the adjusted model. The significance level of 5% was adopted for variables in the adjusted model. *P*-values of the models were derived from the Wald test.

## Results

Between 2000 and 2017, 8,190 children exposed to mother-to-child transmission of HIV during pregnancy were notified. These cases formed the original health surveillance database. Of these, in 520 cases the pregnancy resulted in abortion, in 212 cases the children died within 24 months of follow-up, and in 622 cases moved to a different city where it was not possible to complete the follow-up. Thus, the total for this analysis was 6,836 cases, which corresponded to cases classified as HIV positive, HIV negative, or lost to follow-up. Of the 6,836 cases, 1,763 (25.8%) children were classified as lost to follow-up.

Regarding the completeness of data, there were 928 cases with missing information on race/color. After searching for the information in various medical record systems, 217 cases still had missing information on race/color.

Table 1 describes the characteristics of mothers of lost to follow up. The proportions of mothers of children lost to follow-up differ across age categories (*p* < 0.001). The mothers of children classified as “lost to follow-up” are younger. Regarding race/color, there is a higher percentage of women declaring themselves to be black or mixed in the lost to follow-up group (*p* = 0.001). There is a significant difference in schooling between groups (*p* < 0.001). Pregnant women with up to three years of schooling were more frequent in the lost to follow-up group. Intravenous drug use was more prevalent in the lost to follow-up group (*p* = 0.003). Among women who were HIV diagnosed during prenatal care or at delivery, 34.7% were lost to follow-up, while among those diagnosed before prenatal care, 24% resulted in lost to follow-up (*p* < 0.001). The variable Partner with HIV showed almost 40% loss, so it was not considered for the modeling of associated factors.

**Table 1:** Demographic, behavioral, and health profile of mothers of HIV-exposed children who were lost to follow-up notified to SINAN, in Porto Alegre, according to the outcome lost to follow-up, 2000 – 2017

		Lost to follow-up		
Characteristics	Total (%)^a^	Yes (%)^a^	No (%)^a^	*p* value^b^
	6,836	1,763 (25.8)	5,073 (74.2)	
**Age range**				< 0.001
Up to 22	1,952 (28,6)	574 (29.4)^1^	1,378 (70.6)	
23—26	1,549 (22.7)	409 (26.4)	1140 (73.6)	
27—31	1,691 (24.8)	398 (23.5)	1293 (76.5)	
32 or more	1,640 (24)	383 (23.3)	1258 (76.7)	
**Race/Color**				0.001
White	3,798 (57.4)	917 (24.1)	2,881 (75.9)	
Black or mixed	2,821 (42.6)	782 (27.7)^2^	2,039 (72.3)	
**Schooling**				< 0.001
Up to three years	858 (13.9)	286 (33.3)^3^	572 (66.7)	
Between four and seven years	3,062 (49.7)	796 (26)	2,266 (74)	
Eight years or more	2,244 (36.4)	511 (22.8)	1,733 (77.2)	
**Partner with HIV**				0.149
Yes	3,148 (73.3)	790 (25.1)	2,358 (74.9)	
No	1,148 (26.7)	263 (22.9)	885 (77.1)	
**Intravenous drug use**				0.003
Yes	500 (8.4)	154 (30.8)^4^	346 (69.2)	
No	5,486 (91.6)	1,347 (24.6)	4,139 (75.4)	
**HIV diagnosis**				< 0.001
During prenatal care or at delivery	4,045 (72)	970 (24)	3.075 (76)	
Before prenatal care	1,575 (28)	546 (34.7)^5^	1,029 (65.3)	

^a^Totals may differ due to the possibility of lack of response

^b^Proportion homogeneity test based on Fischer’s Test or Pearson’s chi-square statistic

^1^Standardized residual (= 3.1). ^2^Standardized residual (= 2.2). ^3^Standardized residual (= 4.3). ^4^Standardized residual (= 2.6). ^5^Standardized residual (= 5.9)

Table 2 presents the results of the non-adjusted models and adjusted models age, race/Color, schooling, intravenous drug use, and time of HIV diagnosis. The following socioeconomic, behavioral, and health factors of the studied mothers were associated with lost to follow-up in the final model: being up to 22 years of age (aRR 1.25; 95% CI 1.09–1.43); being self-declared as black or mixed (aRR 1.13; 95% CI 1.03–1.25); up to three years of study (aRR 1.45; 95% CI 1.26–1.67); from four to seven years of study (aRR 1.14; 95% CI 1.02–1.28); intravenous drug use (aRR 1.29; 95% CI 1.12–1.50); and HIV diagnosis during prenatal care (aRR 1.37; 95% CI 1.24–1.52).

**Table 2:** Factors associated with lost to follow-up of children exposed to HIV in Porto Alegre, 2000—2017

	**Unadjusted RR RR**^a^	**CI 95%**	**p**	**Adjusted RR**^b^	**CI 95%**	**p**
**Age range**			< 0.001			0.003
Up to 22	1.26	1.13–1.41		1.25	1.09–1.43	
23—26	1.13	1.01–1.28		1.13	0.97–1.31	
27—31	1.01	0.89–1.14		1.01	0.87–1.17	
32 or more	1			1		
**Race/Color**			0.001			0.013
White	1	-		1	-	-
Black or mixed	1.15	1.06–1.25		1.13	1.03–1.25	
**Schooling**			< 0.001			< 0.001
Up to three years	1.46	1.30–1.65		1.45	1.26–1.67	
Between four and seven years	1.14	1.04–1.26		1.14	1.02–1.28	
Eight years or more	1			1		
**Intravenous drug use**						
Yes	1.25	1.09–1.44	0.001	1.29	1.12–1.50	0.001
No	1	-	-	1	-	-
**HIV diagnosis**						
During prenatal care or at delivery	1.45	1.32–1.58	< 0.001	1.37	1.24–1.52	< 0.001
Before prenatal care	1	-	-	1	-	-

^a^Unadjusted RR estimated by Poisson regression model with robust variation

^b^Adjusted RR estimated by Poisson regression model with robust variation

## Discussion

This study demonstrates the importance of a public database to improve the monitoring of exposed children in a public policy to prevent HIV. The lack of information on the serology status of exposed children within the health care system can impact the planning and implementation of HIV prevention and care [18, 32, 33]. Our data indicate that the epidemiological surveillance system in Porto Alegre is unaware of the diagnosis of about a quarter of the children exposed to HIV. The absence of data also implies possible distortions in the rate of mother-to-child transmission of HIV, as many children considered to have been lost to follow-up may have had a positive result.

This is a problem that affects not only Brazil but also other countries that often have higher rates of loss to follow-up of children exposed to HIV [14, 21, 34, 35]. In a systematic review with meta-analysis [36], the estimated loss of follow-up of children exposed to HIV in 11 Sub-Saharan African countries was 33.9% (95% CI 27.6%-41.5%) in the three-month postpartum period. This study showed that there was little research in Sub-Saharan Africa that included a longer follow-up period. Two of them followed children for 12 months, with lost to follow-up of 50.2% [37] and 85.1% [38]. Other surveillance services that investigated children exposed to HIV for 18 months showed that the loss to follow-up ranged from 20.8 [39] to 66.1% [40]. In a recent study that analyzed the follow-up of children exposed to HIV at a referral hospital in Uganda, the percentage of lost to follow-up was 48% in 18 months [4].

Our data indicate that the loss to follow-up of children exposed to HIV is associated with the different dimensions of the vulnerability of pregnant women with HIV [41]. In the individual dimension, there is intravenous drug use; in the social dimension, being under 26 years of age, being black or mixed and having up to seven years of study; and in the programmatic dimension, the diagnosis of HIV during prenatal care or at delivery. This last result indicates that women with HIV enter the health service later [42, 43].

In the individual dimension, drug use appears to be associated with lower adherence to care for the prevention and treatment of HIV [44, 45]. In addition to the effects of the substances used, marginalization and stigma related to drug use and HIV itself are factors that interfere with access to and adherence to treatment [46]. In this sense, we emphasize that harm reduction strategies [47] could improve care, avoiding loss of follow-up. Care and social support interventions for drug users have shown better standards of care and adherence to drugs [48, 49].

In the social dimension, children whose mothers were up to 22 years old were at an excess risk of 25%, when compared to children of mothers over 32. Age may be interconnected with other factors, such as low socioeconomic status, lower education, and knowledge about HIV [50]. Regarding race/color, black or mixed women had a relative risk 13% higher to have children lost to follow-up. These data can be contextualized from the perspective of institutional racism [51, 52], which accentuates the inequality of access to health which has already been observed in the issue of race/color in Brazil in relation to HIV [7]. Regarding schooling, women with up to three years of schooling had a 45% excess risk of having children lost to follow-up. In the discussion on social vulnerability, education is an important element, as it represents possibilities for entering the labor market and has also been interpreted as an income proxy [53]. In a recent study in Mozambique, higher levels of schooling showed greater chances of having an early start of prenatal care [54]. This study was carried out in a scenario with cultural issues intertwined with the definition of roles associated with gender. We understand that younger women with less schooling may be financially dependent on their sexual partners, which would make the child more vulnerable to being lost to follow-up.

In the programmatic dimension, women diagnosed with HIV during the prenatal period or at delivery had an excess risk of 37% to have a child classified as lost to follow-up. In our study, the percentage of pregnant women who received the diagnosis in prenatal care or at delivery was high and was even greater in the loss to follow-up group (34.7% versus 24%). Barriers to HIV testing in primary care have been documented [55–57], so the possibility of HIV testing during prenatal care appears to be an opportunity to improve care.

This would be an alert for health professionals. The creation of a bond with pregnant women could prevent adverse health outcomes for the child. In fact, in a study carried out in Kenya, knowledge of the diagnosis before prenatal care proved to be a protective factor against loss to follow-up (OR 0.23; 95% CI 0.05–0.71) [18]. A study conducted in the United States found that women diagnosed with HIV during pregnancy are less likely to receive ART adequately during pregnancy and to obtain viral suppression after pregnancy [58].

In our study, the variable moment of diagnosis was divided into two strata: diagnosis during prenatal care or at delivery and diagnosis before prenatal care. Therefore, because the women who may have been diagnosed during prenatal care were grouped with those diagnosed at delivery, we do not know the actual number of women who were diagnosed at childbirth. This is a limitation of our study and is a critical issue to be discussed. Diagnosis at delivery may express the total absence of connection with primary care services or even the failure to perform an HIV test during prenatal care, a recommendation in force in a national protocol. A recent study discussed this issue in Brazil, showing that almost 20% of women in the state of Amazonas reached childbirth without knowing their HIV status, complementing another Brazilian study, in which 29% of women were not tested during prenatal care [59].

From a Brazilian public policy perspective, these results indicate primary care services' difficulty in identifying, linking, and retaining care for children exposed to HIV, problems also seen in other studies [10, 23, 26, 35, 36, 60–63]. In Brazil, primary care services monitor all newborns within their geographic areas of coverage [64]. In a review of access to services to prevent mother-to-child transmission of HIV, Hiarlaithe et al. (2014) identified the following access barriers: cost of traveling to the health service, secrecy in relation to diagnosis, the stigma surrounding HIV, and relationship with the partner [65]. In a study conducted in Ethiopia, it was found that the proportion of pregnant women who have comprehensive knowledge about preventing mother-to-child transmission of HIV was low [47]. The difficulty of access and level of knowledge was not investigated in this study, however, these issues may be related to loss to follow-up.

Like all scientific research, our study is not without limitations. The main one is that our study uses secondary databases, with the researcher having no control over the quality of data collected. However, we emphasize that we have used a greatly valued database by the surveillance team. In addition, the information was complemented by access to other health information systems. Therefore, we believe that this limitation has been reduced. Another limitation of the data already mentioned is the lack of HIV diagnosis during prenatal care or delivery, which prevented us from identifying the percentage of women diagnosed at each of these care moments.

## Conclusion

When considering the sociodemographic, behavioral, and health characteristics of the mothers, we found that the factors associated with loss to follow-up in Porto Alegre are age, race/color, schooling, intravenous drug use, and time of HIV diagnosis. We recommend investing in integrated health information systems so that children exposed to HIV can be located within the health care system, thus reducing the number of losses to follow-up. We also recommend a particular focus on women using intravenous drugs, and the adoption of harm reduction actions, in order to avoid the loss to follow-up of exposed children.

## Data Availability

The datasets used and/or analyzed during the current study is available from the corresponding author upon reasonable request.

## Abbreviations

ART: Antiretroviral therapy
SINAN: Notifiable Diseases Information System
SISCEL: Laboratory Examination Control System
SICLOM: Logistic Control System for Medicines
E-SUS: Electronic medical record of the primary care user
CADSUS: SUS User Registration System
